# The Control of the Invalid Children's Aid Association

**Published:** 1914-02-14

**Authors:** Warrington Haward, Charles W. Freemantle, H. L. Woollcombe, Dorothy H. Carver

**Affiliations:** Chairman, Executive Committee, Invalid Children's Aid Association; Hon. Treasurer, Executive Committee, Invalid Children's Aid Association; Chairman, I.C.A.A. Conference of Branches; Hon. Secretary, I.C.A.A. Conference of Branches


### The Control of the Invalid Children's Aid Association.

Invalid Children's Aid Association.
*(London) Incorporated.*
69 Denison House,
Westminster, S.W.,
February 9, 1914.

The Executive Committee and the Conference of Branches of the Invalid Children's Aid Association desire to repudiate any responsibility for or knowledge of the article which appeared in your paper on January 3 under the heading "The Control of the Invalid Children's Aid Association." They regret that such misleading statements regarding the constitution of the Association, and so inaccurate an account of a confidential meeting should have found its way into your columns.

(Signed)
Warrington Haward, Chairman,
Charles W. Freemantle, Hon. Treasurer,
Executive Committee, Invalid Children's Aid Association.
H. L. Woollcombe, Chairman,
Dorothy H. Carver, Hon. Secretary,
I.C.A.A. Conference of Branches.

[We have received the above typewritten statement dated 9th instant. The document bears no signature, and is simply a typed statement. It is enclosed in a covering letter purporting to come from the Secretary. This also is unsigned, and bears the date of the 7th and not the 9th of February. We have, therefore, no evidence on the face of the documents to prove their authenticity, but we publish the above "paragraph" as received. The article complained of was published in The Hospital of January 3, page 364. Any reader who refers to it will be struck with the fact that it does not contain an "inaccurate" or indeed any "account of a confidential meeting." Of course, as the document accurately states, the authorities of the Invalid Children's Aid Association never had, and never can have, any responsibility for any statement we may publish editorially. The article was favourable and friendly to the Association which we have consistently supported in The Hospital for many years. Any statements deemed to have been misleading should, therefore, as a matter of courtesy, have been quoted and corrected by the authorities. In these circumstances, we are at a loss to decide (*a*) whether our readers and ourselves may properly conclude that the present Executive has lost touch with the Association's progressive development and its importance? or (*b*) whether the documents we have received have been sent without the Executive's knowledge and authority, for some purpose not obvious? The knowledge of experience proves that the executive officers of all properly administered societies write their signatures to official documents they may wish published. Hence our dilemma. We await the official explanation of the mystery.—Ed. The Hospital.]

